# EXCLUSIVE BREASTFEEDING AND UNDERWEIGHT IN CHILDREN UNDER SIX MONTHS OLD MONITORED IN PRIMARY HEALTH CARE IN BRAZIL, 2017

**DOI:** 10.1590/1984-0462/2021/39/2019293

**Published:** 2020-08-10

**Authors:** Thaynara Alves de Miranda Pereira, Agna Kellen Gomes Freire, Vivian Siqueira Santos Gonçalves

**Affiliations:** a Centro Universitário Euro Americano, Brasília, DF, Brazil.; b Universidade de Brasília, Brasília, DF, Brazil.

**Keywords:** Breast feeding, Malnutrition, Children, Brazil, Primary health care, Food consumption, Aleitamento materno, Desnutrição, Crianças, Brasil, Atenção Básica, Consumo alimentar

## Abstract

**Objective:**

To describe the prevalence of underweight and exclusive breastfeeding (EBF) in children aged zero to six months followed by Primary Care in Brazil in 2017, identifying their spatial distribution.

**Methods:**

This was an observational, descriptive and ecological study based on data analysis of the Food and Nutrition Surveillance System. The distribution of records obtained was compared to the population estimates of the Brazilian Institute of Geography and Statistics (IBGE). In order to evaluate the EBF, Primary Health Care teams used food ingestion from the previous day. As for underweight, we used: Length-for-age (L/A), Weight-for-age (W/A) and BMI-for-age (BMI/A), according to World Health Organization (WHO) references. Confidence Intervals were calculated 95% (95%CI) for prevalences obtained, being plotted on maps by Federation Unit.

**Results:**

Data were obtained from 88.7 and 32.2% of Brazilian municipalities regarding anthropometry and food consumption, corresponding to 167,393 and 66,136 children, respectively. Compared to population distribution, the number of records was underestimated in the North and Northeast for anthropometry/consumption, with distinct proportions in the South for anthropometry and Southeast for consumption. The prevalences found were: EBF - 56.6% (95%CI 56.2-56.9); under L/A - 10.6% (95%CI 10.5-10.8); under W/A - 9.0% (95%CI 8.9-9.1); and under BMI/A - 5.8% (95%CI 5.7-6.0).

**Conclusions:**

The estimate of EBF in Brazil was similar to previous studies, but food consumption data still have low coverage, compromising the estimate in some locations. Regarding anthropometry, high rates of low L/A, W/A and BMI/A stood out in some states, considerably above the previous national estimate.

## RESUMO

**Objetivo:**

Descrever as prevalências de baixo peso e aleitamento materno exclusivo (AME) em crianças de zero a seis meses acompanhadas na Atenção Básica no Brasil em 2017, identificando sua distribuição espacial.

**Métodos:**

Tratou-se de um estudo observacional, descritivo e ecológico a partir da análise de dados do Sistema de Vigilância Alimentar e Nutricional. A distribuição de registros obtidos foi comparada às estimativas populacionais do Instituto Brasileiro de Geografia e Estatística (IBGE). Para avaliação do AME, equipes da Atenção Básica utilizaram marcadores de consumo alimentar do dia anterior. Quanto ao baixo peso, usou-se: estatura/idade (E/I), peso/idade (P/I) e índice de massa corpórea (IMC)/idade (IMC/I), conforme referências da Organização Mundial da Saúde (OMS). Foram calculados os intervalos de confiança de 95% (IC95%) para prevalências obtidas, sendo plotadas em mapas, por Unidade da Federação (UF).

**Resultados:**

Obteve-se dados de 88,7 e 32,2% dos municípios brasileiros em relação à antropometria e ao consumo alimentar, correspondendo a 167.393 e 66.136 crianças, respectivamente. Em comparação à distribuição populacional, o número de registros esteve subestimado nas regiões Norte e Nordeste para antropometria/consumo e com proporções distintas na região Sul para antropometria e na região Sudeste para consumo. As prevalências encontradas foram: AME - 56,6% (IC95% 56,2-56,9); baixa E/I - 10,6% (IC95% 10,5-10,8); baixo P/I - 9,0% (IC95% 8,9-9,1); e baixo IMC/I - 5,8% (IC95% 5,7-6,0).

**Conclusões:**

A estimativa de AME no Brasil aproximou-se de estudos anteriores, mas dados de consumo alimentar ainda possuem baixa cobertura, comprometendo a estimativa em algumas localidades. Referente à antropometria, destacaram-se elevadas taxas de baixos E/I, P/I e IMC/I em alguns Estados, consideravelmente acima da estimativa nacional anterior.

## INTRODUCTION

Exclusive breastfeeding (EBF) occurs when the child receives only breast milk, directly from the breast or expressed, without other liquids or solids, with the exception of drops or syrups containing vitamins, oral rehydration salts, mineral supplements or medications. The World Health Organization (WHO), endorsed by the Ministry of Health of Brazil, recommends that breastfeeding should be exclusive for the first six months of life and that it be continued togetherwith complementary foods for up to two years or more.^1^

Childhood is marked by the development of motor, emotional, psychological and social skills. The first years of life are definitive for child development,^2^ and for growth and development to be complete, care must be taken, for example, the provision of EBF.^3^

In the last decades, the prevalence of EBF in children under six months in Brazil has indicated an upward trend, with 34.2% in the period from 1986 to 2006, and 36.6% in 2013.^4^ Children who are breastfed for longer have lower morbidity and mortality from diarrhea, respiratory infections and otitis media, have increased intelligence and protection against being overweight and diabetes in the later life. There are also benefits for breastfeeding mothers, by preventing breast and ovarian cancer and reducing the risk of developing diabetes.^5^

Advances in exclusive breastfeeding practices up to six months and continued breastfeeding up to one or two years could prevent the death of 823,000 children under the age of five and 20,000 women from breast cancer annually, in addition to reducing treatment costs for breast cancer and childhood illnesses of at least $1.8 million in Brazil (equivalent to 2012 dollar quotation). Emphasizing that breast milk is environmentally safe, produced and offered to the child without pollution, packaging or waste.^6^

Adequate weight gain for this age group can also be considered an indicator of good health.^7^ According to the Brazilian National Demography and Health Survey (PNDS ‒ 2006), the last national survey of the infant population, whose parameters for assessing nutritional status of children between zero and 11 months were weight for Height-for-age (H/A) and Weight-for-age (W/A), underweight was present in 2.9% of children, for both indicators.^8^

The monitoring of children under six months of age in Primary Health Care (PHC), in relation to EBF and underweight, can be performed through Food and Nutrition Surveillance (FNS). The implementation of FNS is recommended by the Ministry of Health as a strategy to support the planning of promoting adequate and healthy food consumption in the Brazilian National Health System (SUS), in addition to evaluating implemented actions aimed at good nutrition. One of FNS’s instruments is the Brazilian Food and Nutrition Surveillance System (SISVAN).^9^

The SISVAN is an information system available to the three levels of government (municipal, state and federal), which aims to generate continuous information on the nutritional status of the population. He also works to know its distribution in geographic areas, social segments and population groups at greatest risk to nutritional problems. The system calculates food consumption and anthropometry indicators, in order to monitor the nutritional and food status of the Brazilian population.^3^

The food markers of food consumption in PHC make it possible to investigate the population’s dietary pattern. To this end, a specific module was developed within SISVAN that proposes the assessment of food consumed the previous day, according to WHO guidelines.^10^ In the module for children under six months, the practice of EBF and the early introduction of other foods are evaluated.^11^ In relation to anthropometric assessment, the monitoring of child growth proposed by the Ministry of Health can be performed using SISVAN, by monitoring of weight and height measurements. The WHO recommends the use of specific reference curves to assess the nutritional status of children, and these references are adopted by the system.^12^

 The use of data obtained by SISVAN facilitates the overall observation of the nutritional status and food consumption of children under six months. Although the system provides the population with a lot of information, it is not possible to identify the statistical error estimates inherent to the frequencies presented, nor how to reflect on the proximity of the distribution of cases with the actual distribution of the population across the national territory. Thus, the aim of this study was to describe the prevalence of low weight and EBF in children aged zero to six months followed up in PHC in Brazil in 2017, identifying their spatial distribution.

## METHOD

This was an observational, descriptive and ecological study based on data analysis from SISVAN *web* in 2017, with Brazilian municipalities being the units of analysis. The database was extracted in March 2019, with 2017 being the most recent year and with complete data at that time.

For the assessment of food consumption, the PHC teams used a food consumption marker form, which proposes the assessment of food consumed on the previous day, which can be applied by any professional of the teams. The form evaluates the practice of breastfeeding (BF) and the early introduction of food. Its use must follow the recommendations of the Ministry of Health of 2015, and the registration can be done in instruments, such as medical records, current information form and health handbooks, and later typed in SISVAN.^11^

Regarding the assessment of underweight, the teams collected demographic data (sex and date of birth) and anthropometric data (weight and height). For children under the age of five, it is recommended to use the reference that is already in the Child Health Handbook and SISVAN. The indices and anthropometric parameters used for children in SISVAN and assessed in this study were: W/A, which expresses the relationship between body mass and the child’s age, the index being used mainly to assess the underweight; H/A, which indicates the child’s linear growth, the index that best points to the cumulative effect of adverse situations on growth; and Body mass index-for-age (BMI/A), which explains the relationship between the child’s weight and height status, enabling the assessment of low weight while also considering height. The nutritional assessment of these children was carried out using statistical criteria, using Z score.^13^

Data extraction was carried out by the “Consolidated Reports” module of SISVAN *web* in an aggregated form and without individual identification for assembling the database. After extraction, data on food consumption and nutritional status were compiled in Microsoft Excel for further statistical treatment. Record numbers were compared to population estimates of the Brazilian Institute of Geography and Statistics (IBGE) 2012 with children under one year, with the purpose of detecting the proximity of the distribution between the SISVAN records and what is expected from the population. The 95% confidence intervals (95%CI) were calculated for the prevalence obtained in relation to nutritional status and food consumption for all Federation Units (FU), regions and for the whole of Brazil. Then, the data were plotted on maps by FU for better visual inspection of their distribution. In this study, the software Stata, version 14, and Tab Win, version 4.15, were used for the analyzes.

## RESULTS

In this study, data from 4,945 municipalities were extracted in relation to anthropometry, which corresponds to 88.7% of Brazilian municipalities, and 1,793 in terms of food consumption, equivalent to 32.2%, with 167,393 children being evaluated in relation to anthropometry and 66,136 evaluated in relation to food consumption.

Data obtained by SISVAN were compared to the estimated population data of IBGE, shown in Table 1. The Northeast and North regions had an underestimated distribution of data regarding anthropometry and food consumption. In the Southeast region, on the other hand, the data may have different proportions, mainly with regard to food consumption, indicating an almost doubled percentage when compared to the IBGE. In the South region, the anthropometry reference sample had a different proportion, and in relation to consumption, the sample was similar in terms of population.

**Table 1:** Children under six months of age followed by the Brazilian Food and Nutrition Surveillance System and estimated population in the different Federative Units of the country, according to the Brazilian Institute of Geography and Statistics, 2017.

Regions/States	**SISVAN Sample**		Estimated IBGE population
	Anthropometry	Consumption	
	n (%)	n (%)	n (%)
Mid-West	8,166 (4.8)	3,171 (5.0)	222,617 (7.7)
Distrito Federal	396 (0.2)	221 (0.3)	39,100 (1.3)
Goiás	1,255 (0.7)	90 (0.1)	90,937 (3.2)
Mato Grosso do Sul	4,505 (2.7)	2,256 (3.4)	40,989 (1.4)
Mato Grosso	2,010 (1.2)	604 (1.0)	51,591 (2.0)
Northeast	26,381 (15.7)	4,207 (6.3)	871,471 (30.2)
Alagoas	2,618 (1.5)	749 (1.1)	55,886 (2.0)
Bahia	5,965 (3.5)	793 (1.2)	210,930 (7.3)
Ceará	3,812 (2.2)	724 (1.09)	140,578 (5.0)
Maranhão	1,665 (1.0)	64 (0.1)	131,014 (4.5)
Paraíba	3,464 (2.0)	336 (0.5)	58,736 (2.0)
Pernambuco	4,660 (2.8)	885 (1.3)	137,885 (4.8)
Piauí	1,642 (1.0)	269 (0.4)	50,717 (1.8)
Rio Grande do Norte	1,668 (1.0)	365 (0.6)	49,259 (1.7)
Sergipe	887 (0.5)	22 (0.02)	36,466 (1.2)
North	7,697 (4.5)	2,378 (3.6)	323,649 (11.2)
Acre	431 (0.2)	27 (0.03)	16,245 (0.5)
Amazonas	1,619 (1.0)	176 (0.3)	77,515 (2.7)
Amapá	130 (0.1)	44 (0.05)	15,159 (0.5)
Pará	2,998 (2.0)	1,563 (2.4)	152,996 (5.3)
Rondônia	777 (0.4)	76 (0.1)	26,099 (1.0)
Roraima	233 (0.1)	17 (0.01)	10,097 (0.3)
Tocantins	1,509 (1.0)	475 (0.7)	25,528 (1.0)
Southeast	83,409 (50.0)	48,335 (73.0)	1,091,510 (38.0)
Espírito Santo	1,832 (1.0)	214 (0.3)	50,839 (1.7)
Minas Gerais	55,852 (33.3)	39,257 (59,3)	263,146 (9.1)
Rio de Janeiro	2,594 (1.5)	617 (1.0)	209,325 (7.2)
São Paulo	23,131 (14.0)	8.247 (12.5)	568,200 (19.7)
South	41,740 (25.0)	8,067 (12.1)	370,669 (12.9)
Paraná	19,420 (11.6)	2,335 (3.5)	151,582 (5.2)
Rio Grande do Sul	9,726 (6.0)	3,667 (5.6)	132,369 (4.6)
Santa Catarina	12,594 (7.5)	2,055 (3.1)	86,718 (3.0)

SISVAN: Brazilian Food and Nutrition Surveillance System; IBGE: Brazilian Institute of Geography and Statistics.

Table 2 shows the prevalence rates related to anthropometry, with a focus on identifying underweight by macro-regions and FU. Prevalence was found in Brazil: low H/A - 10.6% (95%CI 10.5-10.8), low W/A - 9.0% (95%CI 8.9-9.1) and low BMI/A - 5.8% (95%CI 5.7-6.0). The State of Minas Gerais had the highest low W/A rate (12.2%; 95%CI 11.9-12.5). With regard to the macro-regions, the Southeast region had the highest rate of low W/A (11.0%; 95%CI 10.8-11.2) and the South region had the lowest prevalences in relation to low W/A (4.3%; 95%CI 4.0-4.5).

**Table 2:** Prevalence rate of low Height-for-Age and low Weight-for-Age in children under six months by macroregions and the Federative Units of Brazil in 2017.

Regions/States	Assessed children	Height-for-Age		Weight-for-Age		BMI-for-Age	
		Low Height-for-Age		Low Weight-for-Age		Low BMI-for-Age	
	n	%	95%CI	%	95%CI	%	95%CI
Brazil	167,393	10.6	10.5-10.8	9.0	8.9-9.1	5.8	5.7-6.0
Mid-West	8,166	9.2	8.6-9.9	6.7	6.2-7.2	5.6	5.1-6.1
Distrito Federal	396	8.6	6.2-11.8	8.1	5.8-112	5.6	3.7-8.3
Goiás	1,255	10.6	9.0-12.4	6.2	5.0-7.7	9.7	8.2-11.5
Mato Grosso do Sul	4,505	9.0	8.2-9.9	7.2	6.5-8.0	4.4	3.8-5.0
Mato Grosso	2,010	9.0	7.8-10.3	5.5	4.6-6.6	5.8	4.9-6.9
Northeast	26,381	11.1	10.7-11.5	4.3	4.0-4.5	1.7	1.6-1.9
Alagoas	2,618	12.5	11.3-13.9	5.6	4.8-6.6	6.0	5.1-6.9
Bahia	5,965	9.5	8.8-10.3	4.0	3.6-4.6	7.2	6.6-7.9
Ceará	3,812	10.4	9.5-11.5	3.4	2.9-4.0	5.4	4.8-6.2
Maranhão	1,665	15.7	14.0-17.5	4.5	3.6-5.6	11.4	9.9-13.0
Paraíba	3,464	10.6	9.6-11.7	4.0	3.4-4.7	4.2	3.6-5.0
Pernambuco	4,660	12.0	11.1-13.0	5.5	4.2-6.2	5.9	5.3-6.6
Piauí	1,642	9.9	8.5-11.4	2.9	2.2-3.8	5.0	4.0-6.2
Rio Grande do Norte	1,668	9.5	8.2-11.0	3.7	2.9-4.7	5.0	4.1-6.2
Sergipe	887	14.1	12.0-16.5	2.9	2.0-4.3	7.0	5.5-8.9
North	7,697	11.0	10.3-11.7	4.9	4.5-5.4	6.5	6.0-7.1
Acre	431	14.2	11.2-17.8	3.5	2.1-5.7	9.1	6.7-12.1
Amazonas	1,619	13.4	11.8-15.2	4.5	3.6-5.6	6.7	5.6-8.0
Amapá	130	16.9	11.5-24.3	3.9	1.7-8.7	6.9	3.7-12.6
Pará	2,998	11.3	10.2-12.5	5.8	5.0-6.7	6.1	5.3-7.0
Rondônia	777	6.7	5.1-8.7	5.7	4.3-7.5	7.3	5.7-9.4
Roraima	233	6.9	4.3-10.9	3.0	1.5-6.1	2.6	1.2-5.5
Tocantins	1,509	9.0	7.7-10.6	4.2	3.3-5.3	6.4	5.3-7.8
Southeast	82,409	11.5	11.3-11.8	11.0	10.8-11.2	6.2	6.0-6.3
Espírito Santo	1,832	9.2	7.9-10.6	8.1	7.0-9.5	7.3	6.2-8.6
Minas Gerais	55,852	13.0	12.7-13.3	12.2	11.9-12.5	6.8	6.6-7.0
Rio de Janeiro	2,594	11.8	10.6-13.1	8.8	7.9-10.0	6.2	5.3-7.2
São Paulo	23,131	8.2	7.9-8.6	8.6	8.2-9.0	4.5	4.2-4.8
South	41,740	8.7	8.4-8.9	9.2	9.0-9.5	4.9	4.7-5.1
Paraná	19,420	8.6	8.3-9.0	9.6	9.2-10.1	4.8	4.5-5.1
Rio Grande do Sul	9,726	8.7	8.1-9.3	10.0	9.3-10.5	5.6	5.2-6.1
Santa Catarina	12,594	8.7	8.2-9.2	8.2	7.7-8.6	4.6	4.2-4.9

BMI: body mass index; 95%CI: 95% confidence interval.

**Figure 1:**
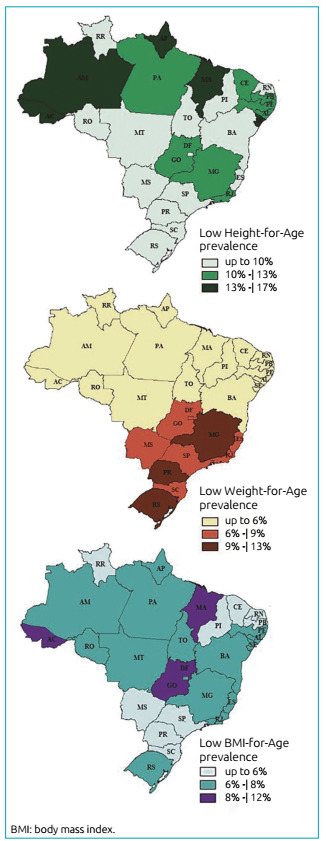
Distribution of inadequacy indicators by Height-for-age, Weight-for-age and BMI-for-age/age index in Brazilian Federative Units in 2017.

**Figure 2:**
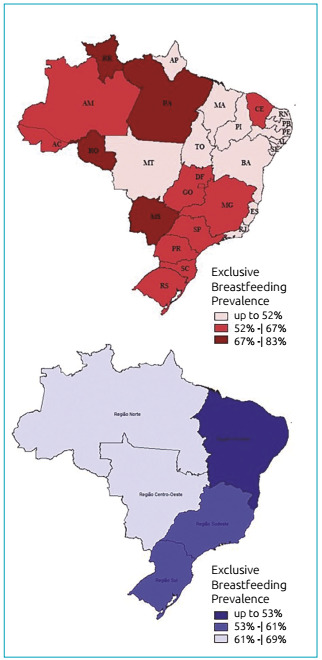
Distribution of exclusive breastfeeding rates in Federative Units and macro-regions of Brazil in 2017.

Table 3 shows the prevalence rates of EBF by macro-regions and FU. The prevalence of EBF in Brazil was 56.6% (95%CI 56.2-56.9). The State of Minas Gerais stood out for the greater number of records and for the more precise prevalence of EBF (54.2%; 95%CI 53.7-54.7). The State of Alagoas had the lowest rate of EBF (40.2%; 95%CI 36.7-43.7). The North region showed the highest frequency of EBF (68.6%; 95%CI 66.7-70.5) and the Northeast region, the lowest (45.9%; 95%CI 44.4-47.4). The prevalences of underweight obtained were plotted on maps, by FU, and EBF, by FU and macroregions, in order to identify their spatial distributions in 2017. Figure 1, the distribution is shown with regard to nutritional status: low H/A, low W/A and low BMI/A. Figure 2 shows the frequency of exclusive breastfeeding in children under six months.

**Table 3:** Prevalence rate of exclusive breastfeeding in children under six months old by macroregions and Brazilian Federative Units in 2017.

Regions/States	Assessed children	Food consumption	
		Exclusive breastfeeding	
	n	%	95%CI
Brazil	66,158	56.6	56.2-56.9
Mid-west	3,171	62.3	60.6-64.0
Distrito Federal	116	52.5	45.9-59.0
Goiás	50	55.6	45.3-65.4
Mato Grosso do Sul	1,522	67.5	65.5-69.4
Mato Grosso	287	47.5	43.6-51.5
Northeast	4,207	45.9	44.4-47.4
Alagoas	301	40.2	36.7-43.7
Bahia	403	50.8	47.3-54.3
Ceará	396	54.7	51.1-58.3
Maranhão	27	42.2	30.9-54.4
Paraíba	138	41.1	35.9-46.4
Pernambuco	366	41.4	38.2-44.6
Piauí	110	40.9	35.2-46.9
Rio Grande do Norte	182	49.9	44.8-55.0
Sergipe	8	36.4	19.7-57.1
North	2,378	68.6	66.7-70.5
Acre	17	63.0	44.2-78.5
Amazonas	101	57.4	50.0-64.5
Amapá	22	50.0	35.8-64.2
Pará	1,234	79.0	76.9-80.9
Rondônia	55	72.4	61.4-81.2
Roraima	14	82.4	59.0-93.8
Tocantins	189	39.8	35.5-44.3
Southeast	48,335	56.1	55.6-56.5
Espírito Santo	111	51.9	45.2-58.5
Minas Gerais	21,292	54.2	53.7-54.7
Rio de Janeiro	306	49.6	45.7-53.5
São Paulo	5,393	65.4	64.4-66.4
South	8,067	59.3	58.2-60.4
Paraná	1,355	58.0	56.0-60.0
Rio Grande do Sul	2,289	62.3	60.7-63.8
Santa Catarina	1,138	55.4	53.2-57.5

95%CI: 95% confidence interval.

## DISCUSSION

The findings of this study update the frequencies of the indicators recommended by the Ministry of Health for the surveillance of EBF and the nutritional status of infants in Brazil. Despite the fact that the distribution of cases sometimes differs from the Brazilian population distribution, according to the IBGE, it was found that the small amplitude of some 95%CI points to the precision of the results and the relevance of using the data generated by SISVAN. Considering that these data are continuous and collected in municipalities that have never been investigated by any other population survey, the value of the system for food and nutritional surveillance in the first months of life is reinforced.

The study also showed that, despite not using the individualized data, or carrying out any cleaning procedure or quality assessment of the bank, which would not be possible with the aggregated data made available to the public by SISVAN, the results for the main outcomes were similar to a previous study that performed such procedures and used individualized data.^14^ These aspects reinforce the importance of aggregated data from SISVAN as a surveillance and management tool for food and nutrition actions in PHC and provide greater security to managers and health professionals regarding the development of interventions which are necessary to promote adequate and healthy eating in their regions.

To be considered good, the prevalence of the EBF indicator should be between 50 and 89%, according to the classification proposed by the WHO.^15^ In the present study, out of all the macroregions evaluated, the Northeast region was the only one that showed a prevalence of EBF below the WHO recommendation. Only the states of Bahia and the Ceará obtained desirable values for classification. The Mid-West, North and South presented a state with lower prevalence: Mato Grosso, Tocantins and Rio de Janeiro respectively. In the Southern region, all states were included in the recommended range.

The prevalence of EBF in Brazil (56.6%) was within the classification of a good indicator; and as a consequence, an early introduction of food was reported for 43.4% of children. The practice of EBF in Brazil is still far from the ideal WHO recommendations (90-100%). However, when compared to the Brazilian National Health Research (PNS - 2013), an increase was found, in view of the 45.4% prevalence in 2013.^4^ This difference, however, may be justified by the fact that the present study only included PHC users, which is a level of health care where actions to promote and protect BF practices are commonly performed.^14^

As for the Brazilian macro-regions, the Northern region presented a higher frequency of EBF (68.6%), which is similar to the study by Wenzel and Souza,^16^ which showed the prevalence of EBF according to socioeconomic and demographic conditions in 2002-2003, with 63% in the North. In this same study, it was found that the prevalence of EBF was higher in rural areas, since the permanence of traditional cultural patterns or the maintenance of family support structures assisted the practice. Another significant aspect is that this practice may be associated with the presence of different structures in the services and health programs. The Northeast region had the lowest EBF rate (45.9%), which was similar to the study by Venâncio et al.,^17^ estimating the practice of EBF in Brazilian capitals and regions in 2008, with a prevalence of 37.0%, the worst situation compared to other regions.

Among the FU, Minas Gerais showed the largest number of records and prevalence of EBF, with good precision, however no studies on EBF for the State were identified. In relation to the capital Belo Horizonte, there was a 39.4% prevalence of EBF in 2008. Sergipe was the state that obtained less frequently in this study (36.4%), and in 2008, the capital Aracaju presented a 34% estimate for EBF.^18^

The frequency of anthropometric indicators (low W/A and low H/A) proved to be increased compared to the PNDS ‒ 2006 with children aged 0‒11 months in 2006, and the deficit prevalence of W/A and H /A were of 2.9 and 4.9%, respectively.

Methodological and age range differences limit the comparison between studies, but highlight the need for further investigation regarding adequate weight and height gain in this age group, in view of the higher values found. The fact that the PHC population is more vulnerable from a socio-economic point of view can also help to explain these discrepancies. The expressive number of children evaluated in the present study (167,393) and the reach of municipalities never represented in population surveys reinforce, however, the value of this estimate.

The Maranhão state had the highest frequency in relation to low anthropometric indicators H/A and low BMI/A. Lopes et al.^19^ indicated the nutritional profile of children under five years of age in Maranhão in 2010, showing that the prevalence of low H/A was 7.7% of the 74 children evaluated, and regarding the low BMI/A index (underweight), the frequency was 1.7% of the 16 children evaluated. This study also showed that the indicator food insecurity in Maranhão has a significant correlation with the nutritional status. Children who live in vulnerable conditions are at greater risk of low H/A.

The Minas Gerais State had the highest rate of low W/A. In line with these data, another study carried out with children aged 0-7 years benefiting from the Bolsa Família Program (PBF) in Minas Gerais , monitored by SISVAN in 2008‒2011, highlighted the prevalence of low W/A in 2011 of 3.0% of the 11,880 children evaluated.^20^ Despite the discording age group, one realizes that in the most vulnerable populations, such as PHC users and PBF recipients, the prevalence of low weight have remained significant over time.

According to a study carried out in a municipality in the Northeast with children under one year old, there are several variants that can influence the practice of BF, such as the number of children, type of delivery and guidance on BF, which demonstrated a statically association in consideration the time when children are breastfed.^21^ Regarding the factors that affect the duration of exclusive breastfeeding, Capucho et al.^22^ conducted a study in Vitória, Espírito Santo, in 2017, which reports that the family context, the previous experience, the psychological, the maternal work and the mammary complications may be related to early weaning. The study also reports that breastfeeding should be encouraged for everyone, and that the mother must feel comfortable about doubts, insecurities and the difficulties in the breastfeeding process.

The present study has limitations because it uses secondary data, assessed nutritional status and food consumption in different numbers of cases, there was an absence of data in some locations in Brazil, in addition to a different year and age range than the IBGE estimate, which decreases the possibility of extrapolating the results achieved. It should also be noted that the sample studied is not representative of Brazilian children, as only those monitored in the PHC were represented, selected for convenience, as in any sample originating from health services. However, it is believed that the results of this study may advance knowledge regarding the frequency of EBF and underweight in Brazil. Regarding anthropometry, high rates of low H/A, W/A and BMI/A were found in certain states, considerably above the national estimate. Despite the limitations, the study brings a robust number of evaluations, allowing for good precision in most of the estimates.

The estimate of EBF in Brazil came close to previous studies, but food consumption data still have low coverage, compromising the estimate in some locations. Through the results obtained reinforces it is necessary to strengthen the actions already implemented and present new actions for the promotion, protection and support of EBF, in order to increase the prevalence and the duration of exclusive breastfeeding, providing adequate weight gain and, consequently, improving the quality of life of children assisted in PHC.
